# Breast cancer or metastasis? An unusual case of metastatic malignant pleural mesothelioma to the breast

**DOI:** 10.1186/s12957-015-0491-z

**Published:** 2015-02-25

**Authors:** Marialuisa Framarino-dei-Malatesta, Paolo Sammartino, Martina Derme, Isabella Iannini, Gabriele Masselli, Irene Pecorella

**Affiliations:** Department of Gynaecological, Obstetrical and Urological Sciences, Umberto I Hospital, Sapienza University of Rome, Viale del Policlinico 155, 00161 Rome, Italy; Department of Surgery “Pietro Valdoni,” Umberto I Hospital, Sapienza University of Rome, Viale del Policlinico 155, 00161 Rome, Italy; Radiology Dea Department, Umberto I Hospital, Sapienza University of Rome, Viale del Policlinico 155, 00161 Rome, Italy; Department of Radiological, Oncological and Anatomical Pathology Sciences, Umberto I Hospital, Sapienza University of Rome, Viale Regina Elena 324, 00161 Rome, Italy

**Keywords:** Malignant pleural mesothelioma, Breast metastasis, Breast cancer

## Abstract

**Background:**

Metastases to the breast from extramammary malignancies are very rare, and ruling out the diagnosis of primary breast tumor is important in order to decide on clinical management and predict prognosis.

**Case presentation:**

Clinical examination revealed in a 49-year-old hairdresser a 3-cm hard lump adherent to the underlying layers in the right breast. Trucut biopsy was performed. Histology showed a solid proliferation of medium-sized neoplastic polygonal cells. Immunohistochemical analysis showed tumor cells diffusely positive for cytokeratin 8/18 and calretinin and focally positive for cytokeratin 5/6 and Wilms’ tumor 1, e-cadherin, and human bone marrow endothelial-1. Estrogen receptors and progesterone receptors were negative. The final diagnosis was metastatic epithelioid malignant pleural mesothelioma.

**Conclusions:**

Immunohistochemistry is an important tool for a conclusive diagnosis of malignant pleural mesothelioma. Owing to the degree of histological and immunohistochemical overlap, a high level of clinical suspicion is essential in order to avoid unnecessary mutilating surgery.

## Background

Breast cancer is the most common malignancy in women, whereas metastases to the breast from extramammary malignancies are very rare, accounting for only 0.43% of all malignant breast tumors [1]. The most common primary tumors metastasizing to the breast are melanoma (29.8%), lung carcinoma (16.4%), gynecological carcinoma (12.7%), intestinal carcinoma (9.9%), leukemia and lymphoma (8.4%), rhabdomyosarcoma (7.3%), and renal cell carcinoma (1.5%) [1]. The time interval between diagnosis of primary cancer and the appearance of breast metastases ranges from 1 to 5 years [2]. Differential diagnosis between primary breast tumor and metastases to the breast from extramammary malignancies is important in order to decide on clinical management and predict expected results and prognosis.

Malignant pleural mesothelioma (MPM) is a rare and aggressive tumor with a median survival of 4 to 12 months, officially recognized as an occupational disease and a signal disease for asbestos exposure [3]. More than 80% of patients with MPM are men [4]. Because the clinical manifestations of MPM are usually nonspecific, diagnosis is often delayed, commonly for as much as 6 months [5]. Pemetrexed- and cisplatin-based chemotherapy is the reference treatment [6].

We report an unusual case of a woman presenting with a breast mass in which trucut biopsy under ultrasound guidance enabled assessment of metastatic MPM.

## Case presentation

A 49-year-old hairdresser was referred to our Breast Unit for suspected right breast cancer. A family history of breast cancer in a first-degree relative was reported. The patient had no symptoms, such as fever, night sweats, cough, and chest pain. Clinical examination revealed a 3-cm hard lump adherent to the underlying layers in the right breast and extending into the axillary tail. Breast sonography showed a solid polylobated mass (3 × 2.5 cm) with heterogeneous echogenicity and no acoustic shadowing behind the tumor. Mammograms were difficult to interpret due to high breast density. Trucut biopsy was performed.

**Figure 1:**
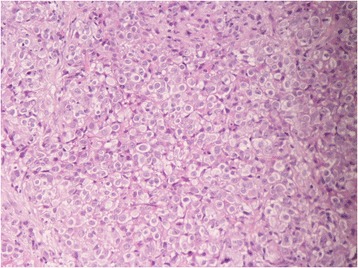
**Hematoxylin and eosin stain (H&E; 96 × 96 dpi).** Infiltrative growth of neoplastic polygonal cells bearing hyperchromatic and pleomorphic nuclei with large eosinophilic nucleolus and a small amount of pale, pinkish cytoplasm.

**Figure 2:**
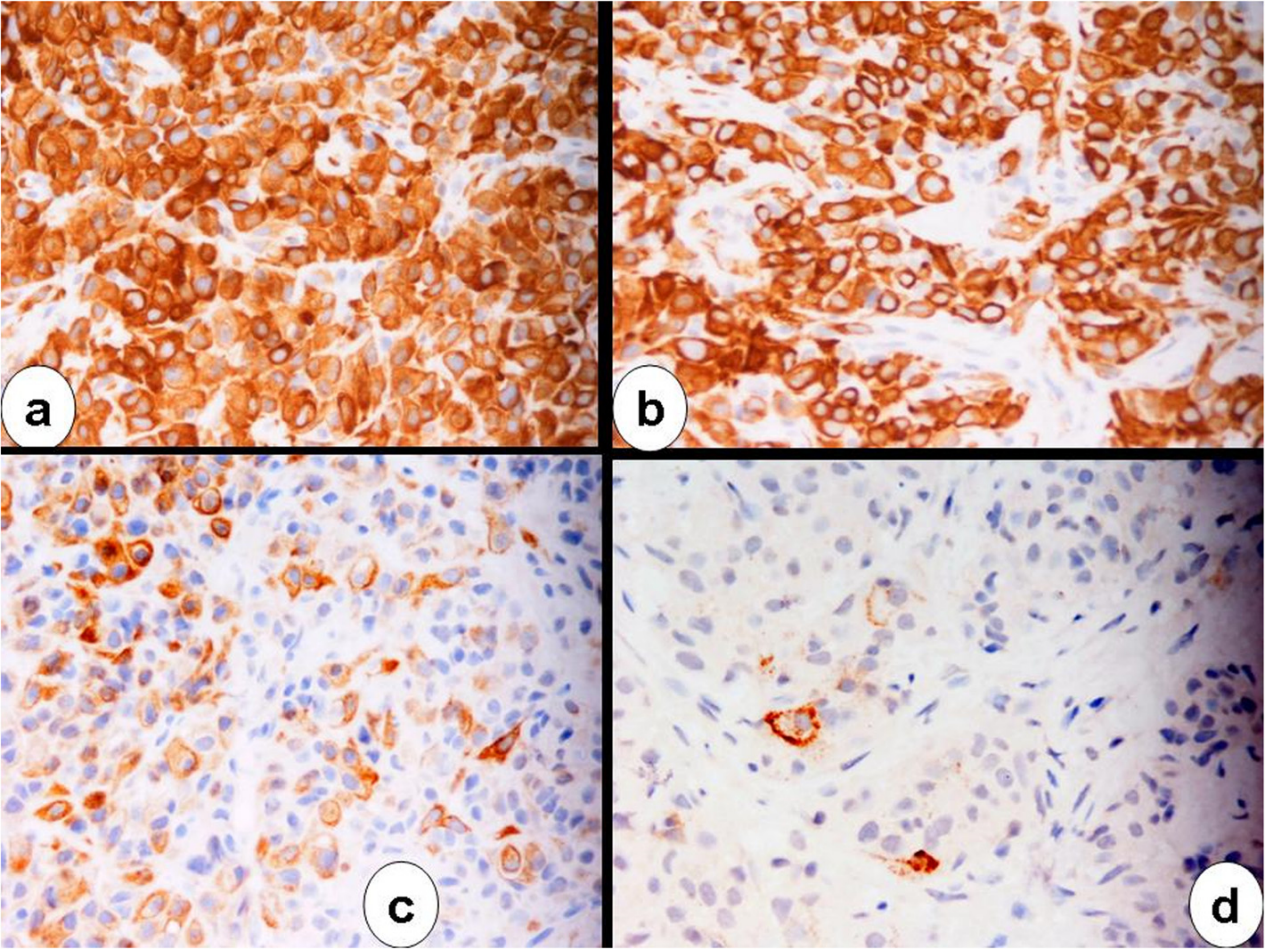
**Immunohistochemical stainings (96 × 96 dpi). (a)** For calretinin, **(b)** for cytokeratin 8, **(c)** for cytokeratin 5/6, and **(d)** for HBME-1 (avidin-streptavidin-peroxidase-DAB).

Histology showed a solid proliferation of medium-sized neoplastic polygonal cells bearing large round vesicular nuclei with central nucleoli and moderate amounts of pale, pinkish cytoplasm. There was little intervening fibrillary stroma and no breast parenchyma (Figure 1). Immunohistochemical analysis showed tumor cells diffusely positive for cytokeratin (CK) 8/18 and calretinin and focally positive for CK 5/6 and Wilms’ tumor 1 (WT1), e-cadherin, and human bone marrow endothelial- 1 (HBME-1) (Figure 2). Estrogen receptors (ER), progesterone receptors (PgR), epidermal growth factor receptor 2 (c-erbB 2), anti-human epithelial antigen (Ber-EP4), thrombomodulin, and carcinoembryonic antigen (CEA) were negative. Proliferation index using Ki-67 was 60%. The final diagnosis was metastatic epithelioid MPM. Upon further questioning, the patient reported a history of daily professional use of asbestos-containing handheld hair dryers.

**Figure 3:**
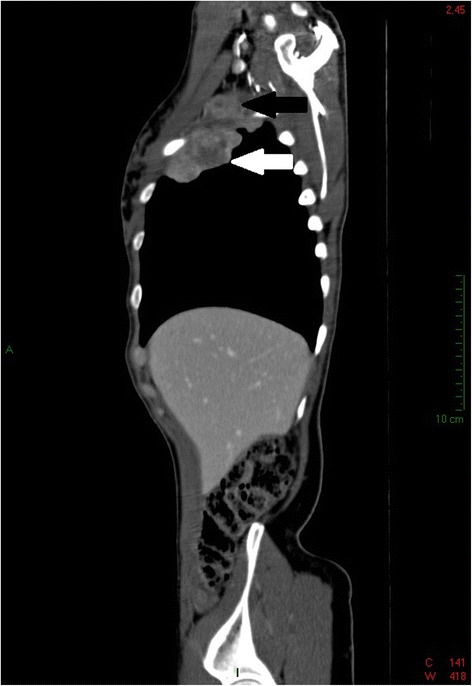
**CT of the chest (96 × 96 dpi).** Sagittal reformatted CT image demonstrates the presence of a parenchymatous neoformation (6 cm) in the right parietal pleura (white arrow) and of another mass in the right axillary extension (3 cm) (black arrow).

In order to confirm the diagnosis and assess thoracic extension, computed tomography (CT) of the chest was performed revealing a parenchymatous neoformation (6 cm) in the right parietal pleura and a lump near the right axilla (3 cm) (Figure 3).

The patient refused conventional cancer treatment preferring alternative therapies. After 9 months, she is still alive.

## Conclusions

Only five cases of metastatic MPM presenting with a breast mass as the initial sign of the disease are reported in the literature, two of them affecting male patients. Ribeiro-Silva *et al*. and Vergier *et al*. described the clinicopathological features of extramammarian malignancies metastasizing to the breast. In both studies, the primary sites of the malignancies included one case of mesothelioma, but breast lesion was not the initial sign [7,8]. Sneige *et al*. reviewed 64 fine-needle aspirates (FNA) performed on the male breast from 1985 to 1992. One case of metastatic MPM was found [9]. Sheen-Chen *et al*. reported a case of a 51-year-old woman with a large pleural mesothelioma and an irregular left breast lesion (4 × 3.5 cm), which proved to be a metastatic deposit. Further immunohistochemical study was performed, and the tumor cells were positive for low and high molecular weight cytokeratin and thrombomodulin and focally positive for CEA [10]. More recently, Aujayeb *et al*. presented a case of a 60-year-old male patient with a right breast mass (13 cm) found to be a metastatic MPM. Immunohistochemistry showed a strong positive expression of calretinin, epithelial membrane antigen, WT1, pan-cytokeratin, thrombomodulin, anti-D240 antibody (D2-40), e-cadherin, and CK 5, 6, and 7 [11].

Breast metastasis was the first manifestation of MPM in our patient. The histopathological appearance of MPM varies and may therefore be a diagnostic challenge, particularly in the presence of a biopsy specimen of an unrelated site. The 2004 World Health Organization (WHO) classification includes three histological subtypes: epithelioid, sarcomatoid, and biphasic mesothelioma. Rare variants are desmoplastic type, undifferentiated type, and deciduoid type. The entirely solid microscopic architecture of the present lesion and the absence of mammary parenchyma in the biopsy specimen raised suspicion of primary non-breast malignancy and prompted further investigation, which led to the correct diagnosis.

Immunohistochemistry is an important tool for a conclusive diagnosis of MPM. The main positive MPM markers include calretinin, WT1, CK 5/6, and HBME-1, whereas gross cystic disease fluid protein 15 (GCDFP-15) and mammaglobin are used to confirm breast cancer, although these markers may be only focally positive depending on the antibody clone used as well as the hormone receptor status of the tumor [12].

As reported in Case presentation section the tumor cells were diffusely positive for calretinin and CK 8/18 and focally positive for CK 5/6, WT1, e-cadherin, and HBME-1.

Calretinin shows strong nuclear and/or cytoplasmic positivity in mesothelial cells and is expressed in epithelioid MPM, but not in the sarcomatoid variant of MPM. However, calretinin expression is not exclusive to MPM [13] as it is expressed in approximately 15% of breast primary tumors, particularly high-grade, ER-negative, and basal-like carcinoma. Consequently, calretinin expression alone could not rule out breast carcinoma in this case.

CK 8/18 is a low molecular weight cytokeratin found in simple epithelium and secretory epithelium. CK 8/18 is markedly upregulated in epithelioid MPM, whereas only ductal *in situ* carcinoma is diffusely positive for this luminal cell marker [14]. Accordingly, the positive result obtained in the present case was useful for ruling out high-grade invasive breast carcinoma.

CK5/6 is a basal cell marker, which is expressed in only 10 to 15% of breast tumors, predominantly in basal-like carcinoma, metaplastic carcinoma, and adenoid cystic carcinoma. In contrast, 75 to 100% of MPMs are CK 5/6-positive [15].

Approximately 75 to 80% of breast tumors are positive for ER and/or PR, but MPMs are not. However, this percentage is reduced to only 2% in breast cancer nuclear grade 3. The present lesion was negative for ER and PgR [16].

Approximately 15% of breast primary cancers of the papillary mucinous subtype are positive for WT1, an independent marker for mesothelioma [17]. However, the present case did not show papillary mucinous differentiation, and WT1 positivity was therefore consistent with a serosal membrane origin of this malignancy.

E-cadherin is normally expressed in the cell membrane of breast ductal cancer cells but not in mesothelial cells. Nevertheless, the present tumor presented focal positive immunostaining, like the case reported by Aujayeb *et al*. [11], thereby hampering its specificity for ruling out MPM.

HBME-1 normally reacts with an antigen present in the membrane of benign and malignant mesothelial cells, and it was focally expressed in our case. However, sensitivity of HBME-1 is high while specificity is low in mesothelial differentiation, as this marker stains various types of adenocarcinoma. HBME-1 is also nonreactive with sarcomatous mesothelioma and with the sarcomatous components of the biphasic variants [18].

Ber-EP4, thrombomodulin, and CEA were negative in this case. Ber-EP4 and CEA are expected to be negative in MPM, but the negativity for thrombomodulin was surprising, considering that the reported sensitivity and specificity of thrombomodulin in epithelioid mesothelioma range from 61% to over 80% [19].

According to the limited data reported in the literature, mesothelioma is immunonegative for both mammaglobin and GCDFP-15. Confirmation of breast origin may therefore be obtained using immunostaining with mammaglobin, GCDFP-15, and androgen receptor in difficult cases, which seem to fit into the triple-negative category of breast carcinoma [20].

In conclusion, as symptomatic metastases of MPM are unusual and metastases to the breast are very rare, clinical and histological misdiagnosis is possible. Owing to the degree of histological and immunohistochemical overlap, a high level of clinical suspicion is essential in order to avoid unnecessary mutilating surgery. A large panel of immunoreactions is necessary to establish a correct diagnosis. We found that CK 8/18, CK 5/6, and WT1 formed a useful panel in this respect, particularly if associated with negative results for ER and PR.

Despite the metastatic disease and the refusal of treatment, this patient is still alive after 9 months, possibly due to the favorable epithelioid histology of the tumor, which is associated with the longest survival (a median overall survival of 16 months).

## Consent

Written informed consent was obtained from the patient for publication of this case report and any accompanying images. A copy of the written consent is available for review by the Editor-in-Chief of this journal.
